# Tail Anchored protein insertion mediated by CAML and TRC40 links to neuromuscular function in mice

**DOI:** 10.1371/journal.pgen.1011547

**Published:** 2025-01-17

**Authors:** Ying Zhang, Lihong He, Justin Gundelach, Anjie Ge, Helena Edlund, Stefan Norlin, Richard J. Bram

**Affiliations:** 1 Department of Pediatric and Adolescent Medicine, Mayo Clinic, 200 1st St. SW, Rochester, Minnesota 55905, United States of America; 2 Mayo Clinic Alix School of Medicine, 200 1st St. SW, Rochester, Minnesota 55905, United States of America; 3 Umeå Centre for Molecular Medicine, Umeå University, SE-901 87 Umeå, Sweden; 4 Department of Immunology, Mayo Clinic College of Medicine, 200 1st St. SW, Rochester, Minnesota 55905, United States of America; The Jackson Laboratory, UNITED STATES OF AMERICA

## Abstract

Motor neuron diseases, such as amyotrophic lateral sclerosis (ALS) and progressive bulbar palsy, involve loss of muscle control resulting from death of motor neurons. Although the exact pathogenesis of these syndromes remains elusive, many are caused by genetically inherited mutations. Thus, it is valuable to identify additional genes that can impact motor neuron survival and function. In this report, we describe mice that express globally reduced levels of calcium-modulating cyclophilin ligand (CAML) protein. CAML is an essential component in the transmembrane domain recognition complex (TRC) pathway, responsible for inserting C-terminal tail anchored (TA) proteins into the endoplasmic reticulum membrane. The primary phenotype observed in these mice was rapid development of hind limb weakness and paralysis. Spinal cord sections revealed a loss of motor neuron cell bodies. Targeting CAML loss specifically to neurons using SLICK-H-Cre or synapsin-Cre transgenic mice yielded similar phenotypes, indicating that CAML plays a cell autonomous role in this process. We found that intracellular trafficking was perturbed in cells depleted of CAML, with aberrant release of procathepsin D and defective retention of CD222 within the trans-Golgi network, as well as reduced levels and mislocalization of syntaxin 5 (Stx5). Dysfunctional lysosomes and abnormal protein glycosylation were also revealed in CAML deficient cells, further indicating a defect in Golgi trafficking. In addition, we observed an identical phenotype in mice lacking ASNA1 in neurons, suggesting that CAML’s role in sustaining muscle function is related to its involvement in the TRC pathway. Together, these findings implicate motor neuron survival as a key role for the TA protein insertion machinery in mice, which may shed light on the pathogenesis of neuromuscular disease in humans.

## Author summary

Many neurological diseases, including neuromuscular disorders, ALS, and Parkinson’s disease, display muscle weakness and paralysis. CAML and TRC35 are two important members in the TRC pathway that facilitate the insertion of a group of proteins called C-terminal tail-anchored (TA) proteins into organelle membranes. Defects in CAML and TRC35 have recently been linked to human neurological disorders. Using newly generated mice with reduced CAML levels, we identified *Caml* as a novel gene required for proper neuromuscular functions in mice. TA protein syntaxin 5 is downregulated and mis-localized when CAML levels are deficient. Lower level of CAML also led to defective membrane trafficking and impaired protein processing in cells. Additionally, mice lacking ASNA1, another member of the TRC pathway, displayed similar neuromuscular disorders to CAML deficient mice. These studies add new insight into the cellular functions of CAML and enhance our understanding of TRC pathway in the pathogenesis of neurological disorders, which may help us to discover better diagnostic strategies and therapeutic targets in patient care.

## Introduction

Motor neurons are among the longest cells in the body, emphasizing their unique dependence on intracellular trafficking [1,2]. Both antegrade and retrograde transport have been shown to be essential for neuron function and survival [3–5]. Pathologic loss or dysfunction of motor neurons underlies a large number of devastating conditions, including genetic diseases like ALS or after acquired insults from toxins such as vinca alkaloids or taxanes [6]. Despite continued research on better understanding the pathogenesis of these motor neuron diseases, there is no treatment for these debilitating conditions. Learning more about the molecular mechanisms that support motor neuron survival in vivo will expand our ability to diagnose neurologic diseases and to discover additional therapeutic opportunities for patients.

Calcium-modulating cyclophilin ligand (CAML) is an integral membrane protein embedded within the lipid bilayer of the endoplasmic reticulum (ER) [4]. As an essential component in the transmembrane domain recognition complex (TRC) pathway, CAML forms a complex with ASNA1 and WRB, facilitating the entry of a class of proteins that have C-terminal transmembrane domains (known as Tail Anchored or TA-proteins) to the ER membrane [7]. Though TA proteins only make up 3%–5% of total integral membrane proteins, they are involved in critical cellular functions including membrane trafficking and protein processing. CAML’s requirement for TA protein insertion may determine its multiple physiologic roles in endocytic trafficking [8], intracellular calcium signaling [9, 10], the suppression of aneuploidy via mitotic spindle function [11], as well as the survival and proliferation of specialized immune cells [12–14].

The TRC pathway consists of conserved proteins (GET1-5 in yeast, and WRB, CAML, TRC40/ASNA1, TRC35, and Ubl4A in higher organisms). Knockout of GET genes in yeast is compatible with life, but negatively influences growth, which could be due to mislocalization of TA proteins [15]. Kao et al identified that loss of ASNA1 in C. elegans impaired insulin secretion, which they propose is its main role [16]. Unlike yeast, genetic knockouts of TA insertion genes are embryonically lethal in higher organisms [11,17,18]. This precludes establishing their roles at the organismal level in the protein processing system. To bypass this limitation, conditional knockouts restricted to specific cell types could allow the identification of relevant functions of components of the TRC40 pathway in tissue development and physiology. Norlin et al engineered cre/lox mice that selectively depleted pancreatic beta-cells of the *Asna1* gene and found that Asna1 deficiency impaired insulin secretion [17]. They further identified an important role for ASNA1 in retrograde trafficking from plasma membrane to trans-Golgi and Golgi to ER. Rivera-Monroy et al likewise selectively knocked out WRB in mice cardiomyocytes or hepatocytes and observed significantly reduced levels of CAML and certain TA proteins [18]. Interestingly, studies using conditional knockouts of both Asna1 and WRB in different cell types revealed that syntaxin 5 is a key TA protein particularly sensitive to loss of the TA insertion machinery.

Like other TA insertion genes, complete loss of CAML results in early embryonic lethality in mice, though embryonic stem cells with deleted *Caml* gene are viable and indistinguishable from wild-type cells [13]. Reduced expression of genes through hypomorphic alleles has been a useful method to identify critical physiological roles of genes. We generated a hypomorphic *Caml* allele by inserting a splice acceptor cassette within an intron, which reduced expression, however, homozygous hypomorphic *Caml* mice do not survive. To further identify CAML’s role in mice, we describe here the creation of a novel hypomorphic model that allowed graded reduction of protein expression globally (termed “pseudo-hypomorphic mice”). To accomplish this, we generated a doxycycline-inducible *Caml* transgenic line (*rtta+*,*Caml-TG+)* and crossed it with *Caml*^*fl/fl*^ mice that carried the ubiquitously expressed, inducible ERT2-Cre transgene (*ERT2-Cre+;Caml*^*fl/fl*^). We added doxycycline to the drinking water to induce low levels of CAML expression from the rtta-dependent transgene, enabling these mice to survive tamoxifen treatment after deletion of the endogenous *Caml* alleles. Here, we show that these pseudo-hypomorphic mice (*rtta+;Caml-TG+;ERT2-Cre+;Caml*^*fl/fl*^) develop lower limb paralysis. We confirmed this finding in *ERT2-Cre+;Caml*^*fl/fl*^ mice exposed to lower doses of tamoxifen that is compatible with the survival as well as in heterozygous *Caml* hypomorphic mice (*Caml*^*+/H*^). Furthermore, we observed similar phenotypes in both *Caml*^*fl/fl*^ and *Asna1*^*fl/fl*^ mice that selectively express Cre recombinase in neurons. Our finding echoed a recent case report on one patient carrying deficient CAML, who presented with severe neurological disorder and psychomotor disability [19].

## Results

### *Caml* pseudo-hypomorphic mice developed hindlimb paraplegia

**Fig 1:**
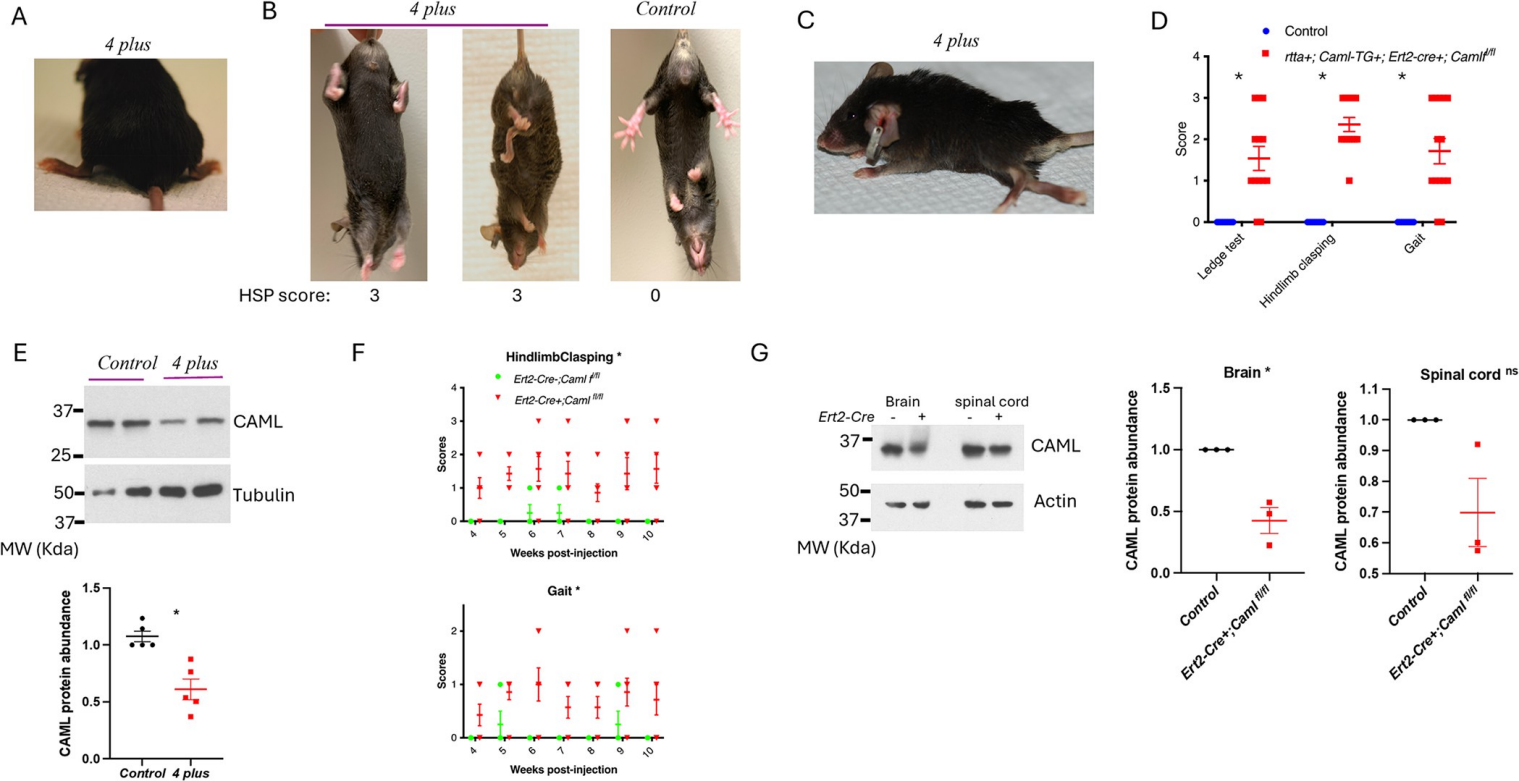
Reduced level of CAML led to hindlimb movement disorders. (A) Representative images of *rtta+;Caml-TG+;ERT2-cre+;Caml*^*fl/fl*^ *(abbreviat*ed to 4 plus) mouse treated with 2 consecutive doses of 75mg/kg tamoxifen displaying a score of 2 on the gait test, where its feet are pointed away from the body during locomotion ("duck feet"). (B) Hindlimb clasping scoring (*abbreviat*ed to HSP score) examples of 3 (first 2 panels) where the hindlimbs are entirely retracted and touching the abdomen. The last panel is score of 0 from a control mouse. (C) Representative image of 4 plus mouse with complete hindlimb paralysis 3 weeks post tamoxifen injections. (D) Scores of the ledge test, hindlimb clasping, and the walking gait 2 weeks post tamoxifen injections. n = 10 for control, n = 14 for *rtta+;Caml-TG+;ERT2-cre+;Caml*^*fl/fl*^ mice received tamoxifen. SEM is shown on the graph, *p<0.*05*, Welch’s t test. (E) CAML level in the spinal cord. The first 2 lanes were from control mice, lane 3 and 4 were from *rtta+;Caml-TG+;ERT2-cre+;Caml* ^*fl/fl*^ *(abbreviat*ed to *4 plus)* mice injected with 2 doses of 75 mg/kg tamoxifen. The quantitative abundance of CAML was normalized to tubulin level and calculated from 5 individuals for each mouse strain. SEM is shown on the graph, *p<0.*05*, Welch’s t test. (F) The dynamic scores of hindlimb clasping (*p<0.*05*, unpaired two-tailed t test) and gait (*p<0.*05*, unpaired two-tailed t test.) in *ERT2-cre+;Caml* ^*fl/fl*^ mice (n = 7) and in *ERT2-*cre-;*Caml* ^*fl/fl*^ mice (n = 8) received a single dose of 25 mg/kg tamoxifen. SEM is shown on the graph. (G) Level of CAML in brain and spinal cord in *ERT2-cre+;Caml* ^*fl/fl*^ mice or *ERT2-cre+;Caml*^*+/+*^ after 1 dose of 25mg/kg tamoxifen. The quantitative abundance of CAML in the brain and in the spinal cord was normalized to actin level and calculated from 3 mice for each strain. SEM is shown on the graph, *p<0.*05*; “ns” is not statistically significant. Unpaired two-tailed t test.

*Caml*^*fl/fl*^ mice that carried the ubiquitously expressed, inducible ERT2-Cre transgene (*ERT2-cre+;Caml*^*fl/fl*^) lived only 3–5 days after 2 consecutive doses of 75 mg/kg tamoxifen. When we started low concentrations of doxycycline (0.2 μg/mL) in the drinking water one week prior to tamoxifen treatment, survival of the pseudo-hypomorphic mice (*rtTA+;Caml-TG+;ERT2-cre+;Caml*^*fl/fl*^) increased to as much as 4 weeks. The most striking phenotype of these mice was the development of hindlimb weakness progressing to paralysis. They first developed characteristic “duck feet” with eversion of the hindlimbs and lowering of the pelvis, indicating loss of strength in the hind quarters (Fig 1A). Meanwhile, they displayed hind leg clasping and retraction into the abdomen (Fig 1B). By 2 to 3 weeks post-tamoxifen treatment, the hind legs were completely extended and paralyzed (Fig 1C). Scores of hindlimb clasping, ledge test, and gait in these pseudo-hypomorphic mice were significantly higher than those in control mice (Fig 1D). Despite expression of the *Caml* transgene, CAML levels were reduced in the spinal cord of *rtta+;Caml-TG+;Esr-cre+;Caml*^*fl/fl*^ mice exposed to tamoxifen (Fig 1E).

We next investigated the effects of reduced doses of tamoxifen on the survival of *ERT2-cre+;Caml*^*fl/fl*^ mice (lacking the rtTA-doxycycline inducible transgene) with limited depletion of CAML. A single injection of tamoxifen at 25 mg/kg did not affect viability. However, at 2 weeks post injection, some *cre+* mice began to develop hindlimb clasping. Scores of hindlimb clasping and gait were recorded weekly from 4 to 10 weeks post tamoxifen injection. Compared to *ERT2-cre-;Caml*^*fl/fl*^ control mice, cre+ mice demonstrated stable rear leg weakness (Fig 1F), though they appeared otherwise quite normal and healthy. Western blotting verified low dose tamoxifen partially reduced CAML protein levels in the spinal cord and brain of *ERT2-cre+;Caml*^*fl/fl*^ mice (Fig 1G). Compared to the unique pseudo-hypomorphic mouse model, *ERT2-cre+;Caml*^*fl/fl*^ mice treated with low dose of tamoxifen had less reduction in CAML protein and presented with milder but similar phenotypes. Taken together, global reduction in two genetic mouse models revealed that CAML is required to preserve strength and function in hindlimbs.

### CAML plays a cell autonomous role in sustaining motor neuron survival in vivo

**Fig 2:**
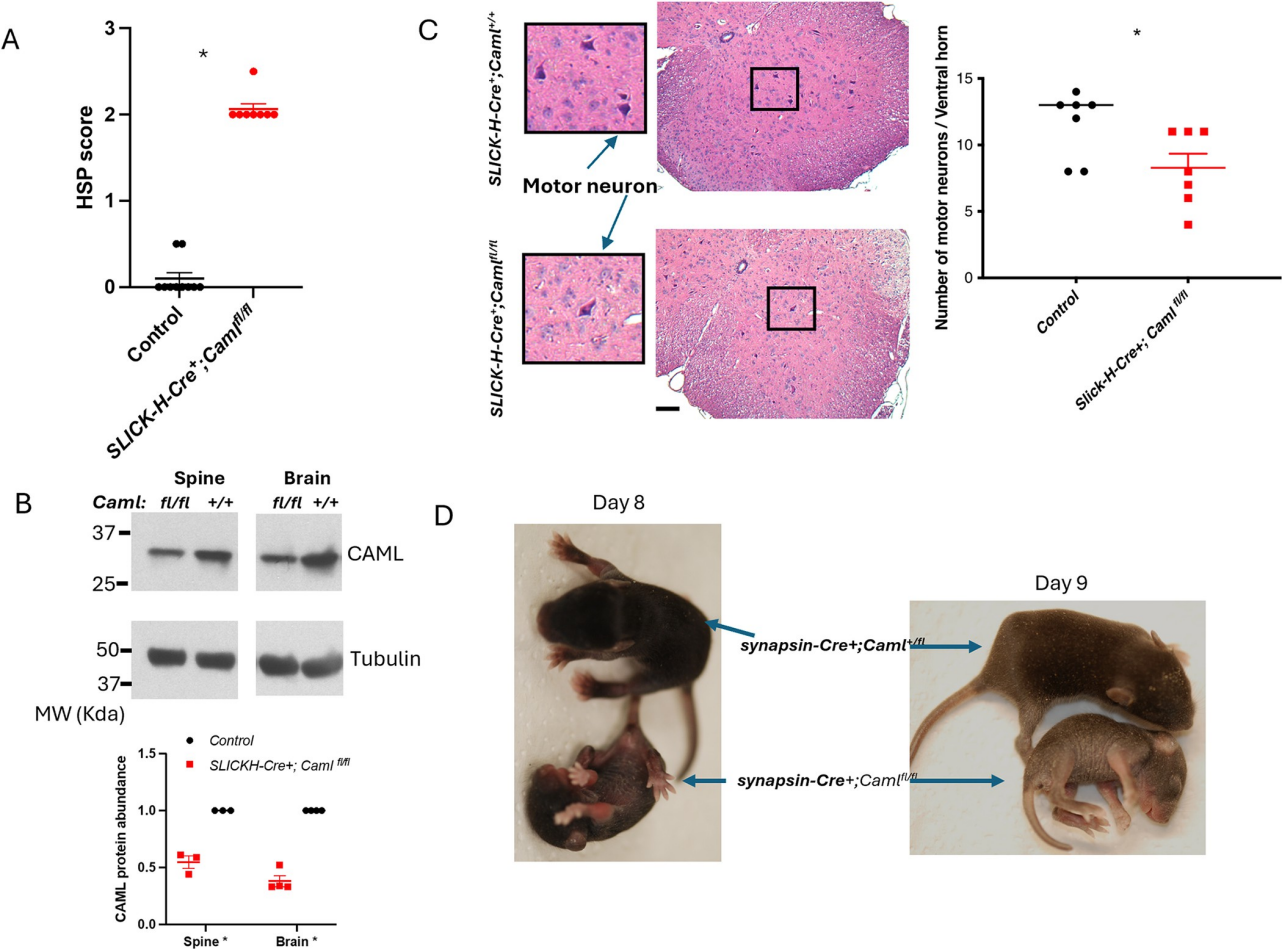
Neuron-specific deletion of CAML induced the development of ataxia and early death. (A) Scores of hindlimb clasping (*abbreviat*ed to HSP score) in *SLICK-H*^*+*^*;Caml*^*fl/fl*^ mice (n = 8) and in control mice including *SLICK-H*^*-*^*;Caml*^*fl/fl*^ or *SLICK-H*^*+*^*;Caml*^*+/+*^ *(*n = 10) 2 weeks followed by 2 doses of 75 mg/kg tamoxifen. SEM is shown on the graph, *p<0.*05*, Welch’s t test. (B) CAML level in the brain and the spinal cord collected from *SLICK-H*^*+*^*;Caml*^*+/+*^ and *SLICK-H*^*+*^*;Caml*^*fl/fl*^ *mice* injected with 2 doses of 75 mg/kg tamoxifen. The quantitative abundance of CAML was normalized to tubulin level and calculated with from 3 mice for spine and 4 mice for brain in each genotype. SEM is shown on the graph, *p<0.05, Welch’s t test. (C) H&E staining of the lumber region (L3-L4) of spinal cord in control and in *SLICK-H*^*+*^*;Caml*^*fl/fl*^ mice 4 weeks post tamoxifen treatment, indicating the motor neurons (100×). Less number of motor neurons were found in lumbar region of spinal cord in mice lacking CAML. *control*: n = 7, *SLICK-H*^*+*^*;Caml*^*fl/fl*^: n = 7. SEM is shown on the graph, Mann-Whitney test, *p<0.*05*. Scale bar = 100 μm. (D) Constitutive expression of Cre recombinase that specifically deletes CAML in neurons induces early postnatal death and malfunction of movement in mice. The mouse in the bottom is *synapsin-Cre+;Caml*^*fl/fl*^ on postnatal day 8 (Bodyweight = 2.8g), its littermate control (top) is 3.8g; on day 9, its bodyweight dropped to 2.5g, the littermate control was 5g. The mouse died on day 10 after birth.

CAML could sustain hindlimb strength through a direct effect on motor neurons or via cell non-autonomous effects. To test this, we crossed *Caml*^*fl/fl*^ mice with SLICK-H-Cre transgenic mice, where Cre recombinase can be induced selectively in neurons. These mice developed hindlimb clasping within one week after treatment with 2 consecutive doses of 75 mg/kg tamoxifen. The measures of hindlimb clasping for SLICK-H-Cre positive were significantly higher compared to SLICK-H-Cre negative mice (Fig 2A). Similar to pseudo-hypomorphic *Caml* mice, mice with reduced CAML protein specifically in neurons (Fig 2B) quickly developed into dramatic paralysis of their rear legs and died within 4 weeks post-tamoxifen treatment. H&E-stained sections on L3-L4 of lumbar spinal cord revealed a 28% reduction in the numbers of motor neuron bodies (Fig 2C).

To ensure that these results were not related to possible off-target effects from tamoxifen, we crossed *Caml*^*fl/fl*^ mice with a constitutive synapsin-Cre transgenic strain, thus expressing the recombinase solely within the neurons without requiring the injection of tamoxifen. Synapsin Cre-positive *Caml*^*fl/fl*^ pups were slightly smaller at birth compared to *Caml*^*+/fl*^ littermates (Fig 2D). As early as neonatal day 5, *Caml*^*fl/fl*^ homozygous pups with Cre had paralysis of the back legs. They could not roll onto their abdomens when they were placed on their backs. Not surprisingly, these mice did not survive past 10 days of age, with significantly loss of hindlimb mobility. These results strongly indicate that CAML serves a cell autonomous role in supporting the survival of motor neurons within the spinal cord.

### Heterozygous *Caml* hypomorphic mice (*Caml*^*+/H*^) developed mild hindlimb paraplegia

**Fig 3:**
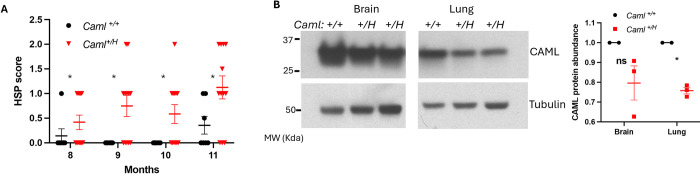
*Caml* hypermorphic heterozygotes developed mild hindlimb clasping. (A) Scores of hindlimb clasping in *Caml*^*+/H*^ mice (n = 7) and in *Caml*^*+/+*^ *(*n = 12). SEM is shown on the graph, *p<0.*05*, Welch’s t test between *Caml*^*+/H*^ group and *Caml*^*+/+*^ *group*. (B) CAML level in the brains and the lungs collected from *Caml*^*+/H*^ (n = 3) and *Caml*^*+/+*^ (n = 2) mice at 6-months-old. The quantitative abundance of CAML was normalized to tubulin level and calculated. SEM is shown on the graph, *p<0.05; “ns” is not statistically significant. Welch’s t test.

As mentioned above, homozygous *Caml* hypomorphic (*Caml*^*H/H*^) mice are embryonically lethal, while heterozygous *Caml* hypomorphic (*Caml*^*+/H*^) mice appear phenotypically normal. Hindlimb weakness observed in the pseudo-hypomorphic mice and in *SLICK-H-Cre+;Caml*^*fl/fl*^ mice led us to revisit these mice carrying only one hypomorphic allele. In the absence of tamoxifen and Cre expression, sporadic *Caml*^*+/H*^ mice developed hindlimb clasping at 3 months of age. In 6-month-old mice, more mice had minor hindlimb clasping. We measured cohorts of *Caml*^*+/H*^ and their wild-type littermates for hindlimb clasping from 8 to 11 months. As shown in Fig 3A, *Caml*^*+/H*^ mice had significantly higher scores than wild-type mice, indicating that they had hindleg disorders, although their phenotypes were mild. There was no difference in ledge test and walking gait between the two cohorts. Brain and lung tissues harvested from *Caml*^*+/H*^ mice had reduced CAML level compared to age-matched wildtype mice (Fig 3B). Overall, limited reduction of CAML level appeared consistent with the mild phenotypes in *Caml*^*+/H*^ mice, which reinforce the previous observations for a physiological role of CAML in the maintenance of neuromuscular function in hindlimbs.

### CAML loss impairs retrograde transport in MEFs

**Fig 4:**
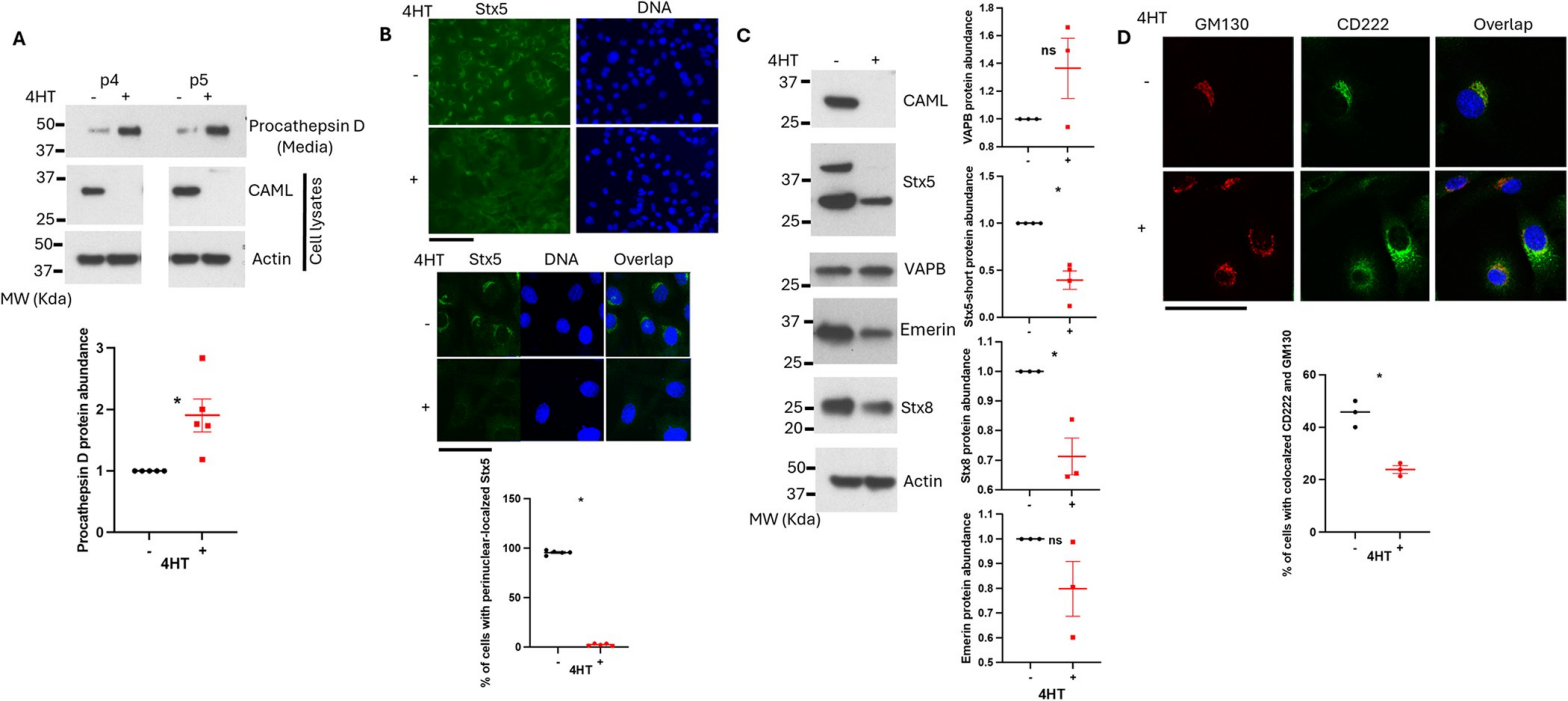
CAML deficiency impairs retrograde transport in primary MEFs. (A) Procathepsin D level in the media culturing *Ert2-cre+;Caml* ^*fl/fl*^ MEFs in the presence or absence of 4HT and the levels of actin, CAML in the corresponding cell lysates. The quantitative abundance of procathepsin D was calculated by procathepsin D level in the media normalized to actin level in the cell lysates using 5 independent MEF lines. SEM is shown on the graph, *p<0.*05*, Welch’s t test. (B) Immunostaining of primary *Ert2-cre+;Caml* ^*fl/fl*^ MEFs treated with or without 4HT. Stx5 (green), Nuclear (blue), 400 X (Top) and 1000 X oil (Bottom). Scale bar = 100 μm. The concentrated accumulation of Stx5 in the perinuclear space was evaluated using 4 independent MEF lines. *p<0.*05*, Welch’s t test. (C) Levels of Stx5, VAPB, Emerin, Stx8 in primary MEFs lacking CAML. The quantitative abundance of Stx5-short was normalized to actin level and calculated using 4 independent MEF lines. The quantitative abundance of VAPB, Emerin, and Stx8 was normalized to actin level and calculated using 3 independent MEF lines. SEM is shown on the graph, *p<0.*05*; “ns” is not statistically significant. Welch’s t test. (D) Immunostaining of primary *Ert2-cre+; Caml* ^*fl/fl*^ MEFs (passage 5) treated with or without 4HT. CD222 (green), GM130 (red), Nuclear (blue), 1000 X. Scale bar = 100 μm. SEM is shown on the graph, *p<0.*05*, Welch’s t test.

We previously showed that *Caml* knockout ES cells had impaired recycling of EGFR when bound by ligand [13]. Norlin et al described the TA insertion mechanism upstream of plasma membrane-to-*trans* Golgi network and Golgi-to-ER retrograde transport [17]. Since retrograde transport has been proposed as an important process in neuron survival, we asked whether *Caml* knockout likewise has an impact on this intracellular process. Primary *ERT2-cre+;Caml*^*fl/fl*^ MEFs are viable in culture, even after total loss of CAML following 4-hydroxy-taxmoxifen (4HT) exposure. However, cells lacking CAML grow slower than wild-type counterpart [11]. If these cells had defective retrograde transport, there would be an expected increase in release of procathepsin into the cell media, instead of delivery to the lysosomes. As predicted, CAML-depleted MEFs showed significantly greater amounts of precursor cathepsin D (procathepsin D) released from serum-starved cells, compared to control cultures (Fig 4A). Procathepsin D level was normalized by equal expression of β-actin in starved MEFs (Fig 4A). Cells maintained survival under these conditions, ruling out cell death related breakage as the cause of increased medium-containing procathepsin.

Pancreatic cells deficient in TA insertion due to the loss of ASNA1 displayed severely altered localization of the TA-soluble N-ethylmaleimide-sensitive factor attachment protein receptors (SNARE) syntaxin 5 (Stx5) and syntaxin 6 (Stx6), which recycle membrane proteins within the cell[17]. Reduced Golgi-localized Stx5 was also reported in the fibroblasts derived from the patient carrying mutant CAML[19]. Our immunofluorescence microscopy revealed that the localization of residual Stx5 was dramatically altered upon *Caml* gene deletion. WT MEFs were presented with the concentrated accumulation of Stx5 in the perinuclear space (Top panels of 400 X and 1000 X, Fig 4B). With almost full knockout of CAML in MEFs, the majority lost the distinctive concentrated Stx5 staining next to the nuclei (Lower panels 400 X and 1000 X, Fig 4B). In addition, we found significantly reduced levels of TA insertion client proteins Stx5 and Stx8, but not emerin and VAPB in *Caml* knockout MEFs (Fig 4C).

The cation-independent mannose 6-phosphate receptor (CD222) is a central component of endosomal transport, as it is required for delivery of lysosomal hydrolases to the lysosome, during which it recycles from the *trans*-Golgi network (TGN) to endosomes. We asked whether disruption of syntaxins downstream of CAML-depletion might impact CD222, given the altered delivery of procathepsin D seen in the knockout MEFs. Through the immunofluorescence staining of primary *ERT2-cre+;Caml*^*fl/fl*^ in the absence and presence of tamoxifen, we observed that knockout of CAML caused significantly more diffused accumulation of CD222 near the TGN, identified by GM130 staining (Fig 4D). Taken together, these results provide evidence that loss of CAML leads to impairment of retrograde transport.

### CAML deficiency leads to dysfunctional lysosomes and defective O-linked glycosylation in primary MEFs

**Fig 5:**
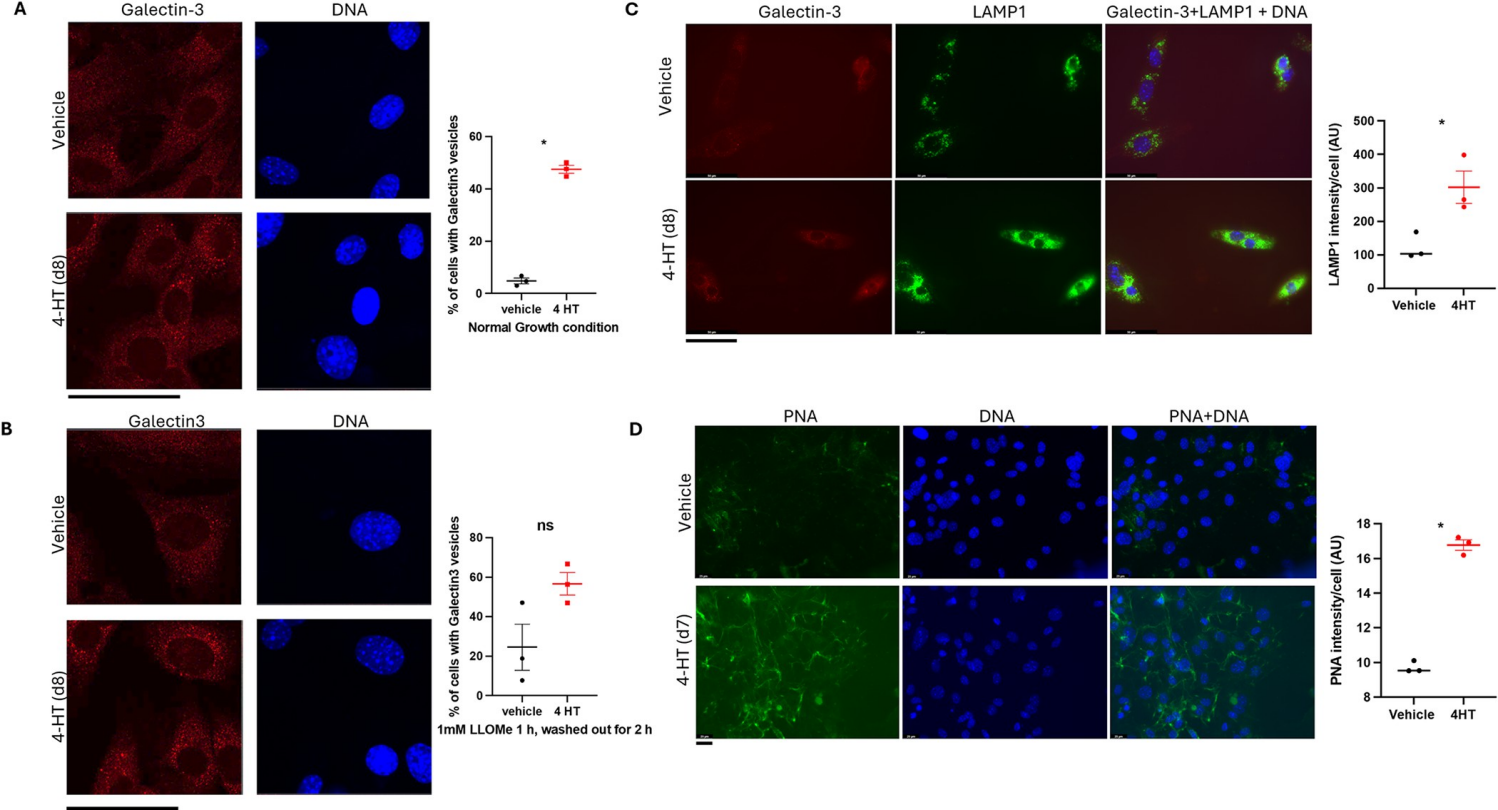
Defective lysosomes and O-glycan sialylation were observed in primary MEFs lacking CAML. (A) Galectin-3 puncta assay in 3 independent primary *ERT2-cre+;Caml* ^*fl/fl*^ MEFs (p6) exposed to vehicle or 4HT. SEM is shown on the graph, *p<0.*05*, Welch’s t test. (630 X) Scale bar = 100 μm. (B) Galectin-3 puncta assay in 3 independent primary *ERT2-cre+;Caml* ^*fl/fl*^ MEFs treated with 1 mM LLOMe for 1 h, and then 2 h washout in the presence or absence of 4HT. SEM is shown on the graph, *p = 0*.*09*, “ns” is not statistically significant. Welch’s t test. (630 X) Scale bar = 100 μm. (C) Co-staining of galectin-3 and lysosomal marker LAMP1 in the MEFs received the same treatment as panel B. The intensity of green fluorescence representing the LAMP1 signals using 3 independent primary *ERT2-cre*^*+*^*;Caml* ^*fl/fl*^ MEF lines was quantified by Image J. AU = artificial unit. *p<0.*05*, Welch’s t test. (400 X) Scale bar = 50 μm. (D) Increased peanut agglutinin (PNA) staining in primary MEFs lacking CAML. Image J was used to evaluate the intensity of green fluorescence that indicates the PNA signals in 3 independent primary *ERT2-cre*^*+*^*;Caml* ^*fl/fl*^ MEF lines. *p<0.*05*, Welch’s t test. (400 X) Scale bar = 25 μm.

Lysosomes play an important role in maintaining neuronal homeostasis. Several neurodegenerative disorders, including ALS, Parkinson’s disease (PD), and frontotemporal dementia (FTD), have been linked to lysosomal dysfunction [20]. The precursor of cathepsin D, a lysosomal protease responsible for the degradation of various disease-associated substrates, abnormally accumulated outside of cells when CAML was absent (Fig 4A). This finding led us to evaluate the lysosomal function in *Caml* knockout MEFs. We adopted the galectin puncta formation assay that detects individual permeabilized lysosomes by a punctate staining pattern to assess lysosome damage [21]. Briefly, we immunostained MEFs using galectin-3 antibody and counted cells with galectin-positive-puncta that represent damaged lysosomes. To minimize the effect of a false-positive background, only cells with greater than 3 puncta were counted into the galectin-3 positive population. There were 8 times more cells with positive galectin-3 puncta in *Caml* null MEFs than in their wildtype counterpart (Fig 5A). This result suggests that loss of CAML indeed induced damaged lysosomes. To verify the galectin-3 puncta assay truly recognizes dysfunctional lysosomes, we performed the same assay on cells exposed to the lysosomotropic reagent L-Leucyl-L-leucine methyl ester (LLOMe). As expected, the number of cells with positive galectin-3 puncta in both wildtype and null background went up, compared to their corresponding number under normal growth conditions. Again, more null cells appeared to express positive galectin-3 puncta in response to LLOMe (Fig 5B). Because galectins also accumulate on permeabilized phagosome and endosomes, we therefore co-stained galectin-3 with lysosomal marker LAMP1 to ensure the observed galectin puncta co-localized were lysosome-related. The result (Fig 5C) confirmed the co-localization of galectin-3 and LAMP1. Strikingly, there were significant enlargements of LAMP1-positive structures in *Caml* null cells. These abnormalities in lysosomal structure further suggest its malfunction in the absence of CAML.

Congenital disorder of glycosylation (CDG) has been linked to clinical cases with deficient CAML and Stx5 short form (Stx5S) [19]. Given that the loss of CAML led to reduced and mislocalized Stx5, we next investigated glycosylation defects in *Caml* knockout MEFs. We stained MEFs with the peanut agglutinin (PNA) lectin that recognizes O-glycans containing terminal galactose-*β*(1–3)-*N*-acetyl galactosamine (Gal-*β*(1–3)-GalNAc). There was a significantly elevated level of PNA lectin fluorescence in *Caml* null cells compared to control cells (Fig 5D). As the increased level of PNA binding indicates hyposialylation of O-glycans, which is commonly seen in other CDG, our result suggests that sialylation may be deficient in the cells lacking CAML. Since many proteins with improper glycosylation, including procathepsin D, are not secreted and targeted to the lysosomes, defective glycosylation may directly contribute to abnormal lysosomal function when CAML is absent.

### Less severe phenotypes are associated with CAML loss in embryonic cortical neurons

**Fig 6:**
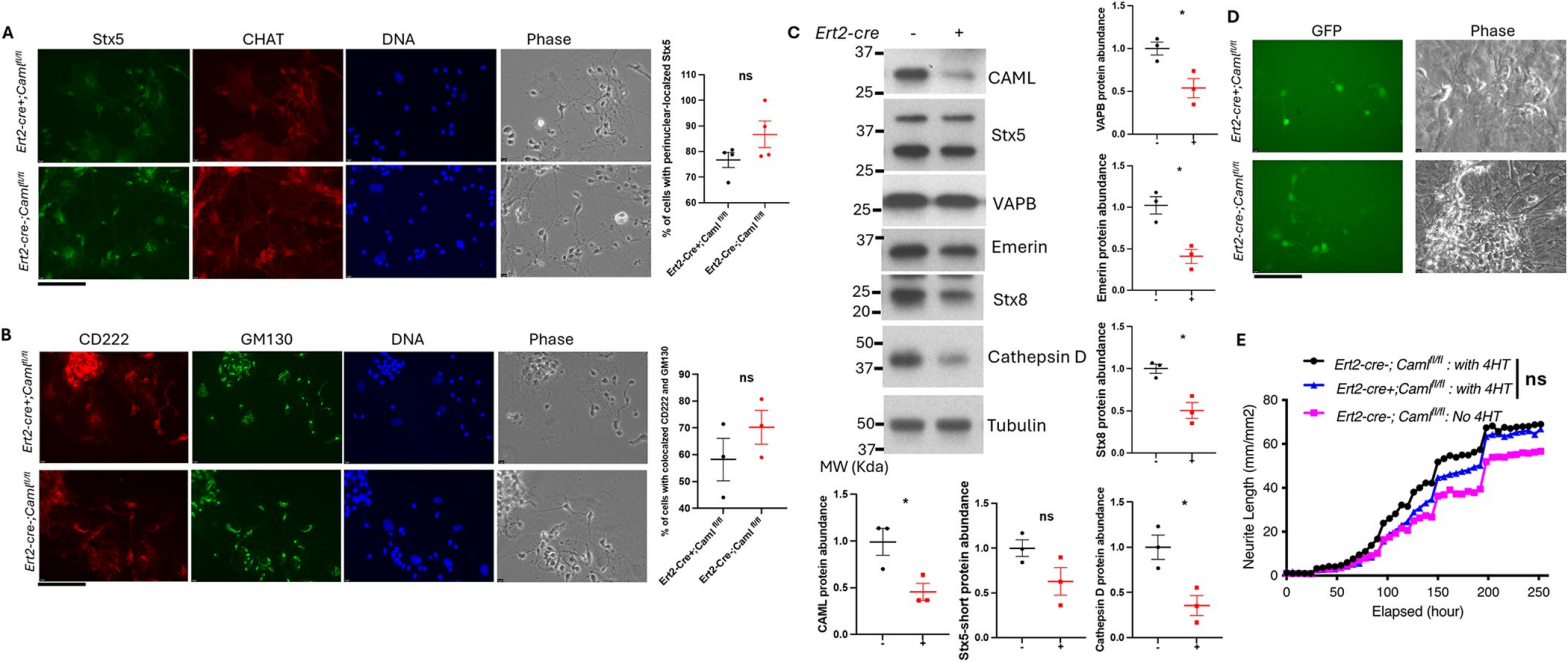
Less profound phenotypes were observed in primary cortical neurons than those in MEFs. (A) Immunostaining of primary cortical neurons generated from day 18 embryos. Stx5 (green), CHAT (red), Nuclei (blue), 400 X. Scale bar = 100 μm. The number of perinuclear-localized Stx5 from 3 lines of *Ert2-cre-;Caml* ^*fl/fl*^ and 3 lines of *Ert2-cre+;Caml* ^*fl/fl*^ was counted and analyzed. “ns” is not statistically significant. Welch’s t test. (B) Immunostaining of primary cortical neurons. CD222 (red), GM130 (green), Nuclei (blue). The colocalization of CD222 and GM130 in primary neurons from 3 lines of *Ert2-cre-;Caml* ^*fl/fl*^ and 3 lines of *Ert2-cre+;Caml* ^*fl/fl*^ was analyzed. “ns” is not statistically significant. Welch’s t test. 400 X. Scale bar = 100 μm. (C) Levels of Stx5, VAPB, Emerin, Stx8 and Cathepsin D in primary cortical neuron cells depleted CAML. *Ert2-cre-;Caml*^*fl/fl*^: n = 3; *Ert2-cre+;Caml*^*fl/fl*^: n = 3. The quantitative abundance of Stx5-S, VAPB, Emerin, and Stx8 was normalized to tubulin level and calculated. SEM is shown on the graph, *p<0.*05*, “ns” is not statistically significant. Welch’s t test. (D) Primary cortical neurons were identified by GFP signals. 400 X. Scale bar = 100 μm. (E) GFP+ cortical neurons with reduced CAML appeared normal in growth and neurite length. 4HT is 4-hydroxl tamoxifen (*Ert2-cre-;Caml* ^*fl/fl*^: n = 3; *Ert2-cre+;Caml* ^*fl/fl*^: n = 5). “ns” is not statistically significant. Welch’s t test between group of *Ert2-cre-;Caml* ^*fl/fl*^ with 4HT and group of *Ert2-cre+;Caml* ^*fl/fl*^ with 4HT.

Unlike MEFs, neuron growth is dependent on multiple types of cells, including astrocytes, microglia and oligodendrocytes, which may respond to tamoxifen differently. The CAML level was significantly reduced in neuronal cultures following tamoxifen treatment, however, to a lesser degree than what was observed in MEFs (Fig 6C). Along with reduced CAML, the levels of TA insertion client protein Stx5 didn’t decrease significantly, though trending to be less. However, three other client proteins, Emerin, VAPB, and Stx8 had statitically significant reduction after *Esr-cre+;Caml*^*fl/fl*^ neuron culture was exposed to 4-HT (Fig 6C). We identified neurons under bright field and with choline transferase (CHAT) co-staining. Subcellular localization of Stx5 and CD222 appeared in a similar fashion to knockout MEFs, though the alterations were mild and not significant (Fig 6A and 6B).

Previously, we showed that aberrant trafficking of cathepsin D occurs in *Caml* knockout MEFs (Fig 4A). As an important player in lysosomal recycling and degradation of many substrates, cathepsin D is highly expressed in the brain. Multiple genetic variants within the *CTSD* gene have been linked to neurodegenerative diseases [22]. Patients with significantly reduced Cathepsin D activity presented with ataxia and progressive psychomotor retardation. Given the phenotype observed in *Caml* hypomorphic mice, we asked if CAML deficiency targeted cathepsin D. In comparison to control cortical neuron cultures, cathepsin D levels were significantly reduced when *Caml* was deleted (Fig 6C). This result implicates that retrograde transport in cortical neurons is less effective when CAML level was reduced.

*Caml* hypomorphic mice had fewer motor neurons in the lumbar region of the spinal cord, leading us to exploit the embryonic cortical neuron cultures to test if CAML deficiency similarly interferes with primary neuron survival. Cortical neuron cultures were infected with a synapsin-GFP expressing adenovirus prior to 4HT treatment and were tracked by Incucyte for 10 days after tamoxifen was added. We specifically monitored neuron development with GFP signals. Neurons with insufficient CAML appeared be normal and healthy (Fig 6D). Using Incucyte neurotrack software, we examined several features of neurite outgrowth and branching. There was no significant difference between length and branch points of neurites, regardless of presence or absence of the *Caml* gene (Fig 6E). These results in embryonic cortical neurons certainly argue against the consistent phenotypes seen in whole-body or neuron-specific *Caml* hypomorphic mice. The *in vitro* culture conditions for primary neurons and the communication among different cell types in cortical neuron cultures may not be capable of mimicking the development of motor neurons *in vivo*.

### ASNA1 loss in neurons causes hindlimb paralysis in mice

Lastly, we asked if hindlimb paralysis phenotype reflects a specific role for CAML in supporting neuromuscular function or if TA protein insertion is involved. To test this, we used mice carrying two loxP sites flanking exon 2 of *Asna1*, the other essential member of the TRC pathway that delivers TA proteins to the CAML/WRB receptor complex on the ER. We crossed *Asna1* flox (*Asna1*^*fl/fl*^) mice to SLICK-H-cre transgenic mice to generate neuron-specific *Asna1* conditional knockout mice. Previous studies in *Asna1*^*fl/fl*^ mice that express β-cell specific-CRE recombinase showed significantly decreased *Asna1* gene expression and protein levels in the presence of CRE [17].

**Fig 7:**
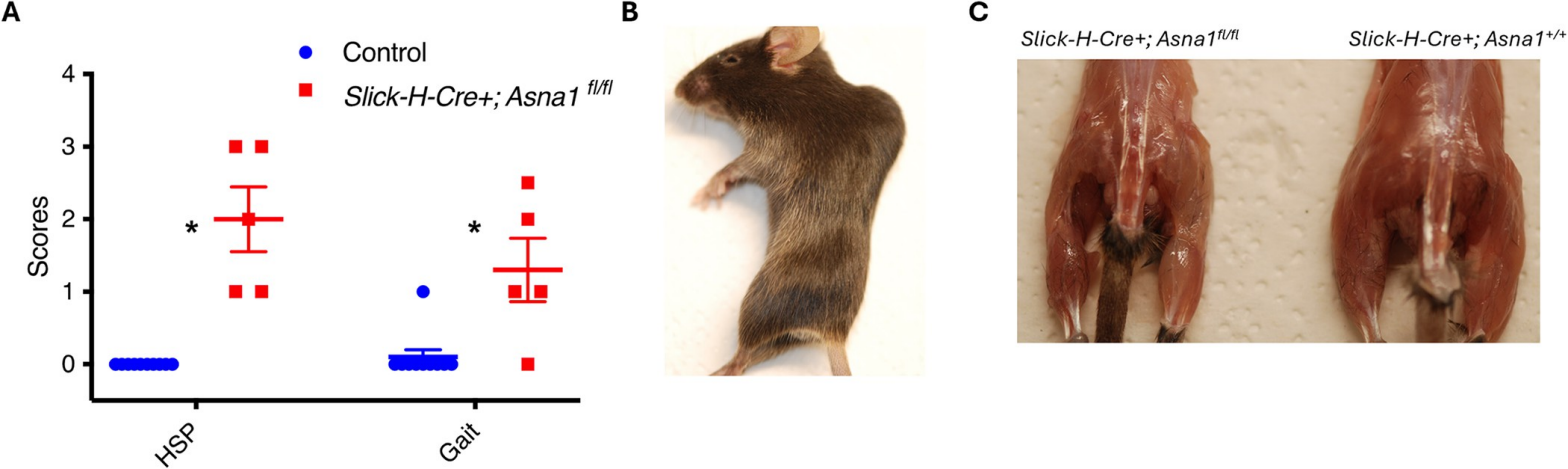
Neuron-specific deletion on Asna1/TRC40 also induces the paralysis of hindlimbs in mice. (A) Scores of the hindlimb clasping, and the gait 3 weeks after receiving one dose of 75 mg/kg. n = 10 for control, n = 5 for *Slick-H-Cre+;Asna1*^*fl/fl*^ mice. SEM is shown on the graph, *p<0.*05*, Welch’s t test. (B) Representative image of *Slick-H-Cre+;Asna1*^*fl/fl*^ 4 weeks post tamoxifen injection. (C) Reduced muscle mass on hind legs in *Slick-H-Cre+;Asna1*^*fl/f*^ mice (Left) 4 weeks post tamoxifen injection.

Interestingly, we observed similar results to our *Caml* hypomorphic animals described earlier. *SLICK-H-Cre+;Asna1*^*fl/fl*^ mice rapidly developed hindlimb weakness, progressing to paralysis and reduced locomotion after receiving one dose of 75 mg/kg tamoxifen. We scored hindlimb clasping and gait at 3 weeks post-tamoxifen treatment. *SLICK-H-Cre+;Asna1*^*fl/fl*^ mice presented with significantly higher scores than control mice (Fig 7A). Four weeks following the same dose, *SLICK-H-Cre+;Asna1*^*fl/fl*^ mice were completely paralyzed (Fig 7B). Along with dramatically reduced muscle mass (Fig 7C), their hind legs lost all mobility. The striking phenotypic resemblance in *Asna1* and *Caml*-deficient mice indicates that the TRC pathway provides critical support for motor neurons and sustains limb strength in mice.

## Discussion

In this study, we uncovered an important physiological role for CAML in mice is to support hindlimb muscle innervation by sustaining the survival of spinal cord motor neurons. Although prior reports from our lab and others have performed tissue specific knockout of the *Caml* gene using Cre/lox technology [11–13], this is the first report that examines the impact of reduced CAML expression within the entire organism by constructing a pseudo-hypomorphic mouse model with protein levels reduced globally in multiple tissues. This approach was necessitated by the embryonic lethality of complete knockout mice and the inability of a standard hypomorphic strategy to produce viable mice. In this approach, we used an inducible *Caml* transgene paired with inducible total body deletion of endogenous *Caml* expression to enable more finely tuned control of protein expression. Although the generation and breeding of these pseudo-hypomorphic mice is more involved than traditional hypomorphic alleles that utilize insertion of a cryptic splice acceptor within an intron, this new approach may be valuable in the analysis of other genes that cannot otherwise be conveniently studied due to embryonic lethality.

Upon identifying a key role for CAML in neurons, we used the minimal viable dose of tamoxifen for *ERT2-cre+;Caml*^*fl/fl*^ mice without the rtTA-doxycycline inducible *Caml* gene. These mice consistently presented with similar hindlimb phenotypes as previous pseudo-hypomorphic mice. We also used SLICK-H-Cre, a more focused mouse model that allows for the inducible deletion of *Caml* genes specifically within neurons. As in pseudo-hypomorphic *Caml* mice, these mice were also impacted by tamoxifen treatment, resulting in rapid paralysis of hindlimbs. These findings provided strong evidence for a cell autonomous role for CAML in neurons. Additionally, we tested the impact of constitutive deletion of CAML in neurons using the non-inducible synapsin-Cre transgene, and again identified the dramatic loss of hind leg control in newborn pups. Embryonic development did not seem to be detectably impaired in these *synapsin-Cre+;Caml*^*fl/fl*^ pups, although they died 10 days after birth. Together with the mild hindlimb paraplegia observed in *Caml*^*+/H*^ mice that we revisited, any potential contribution of off-target effects from tamoxifen were ruled out.

Currently, there are only a few identified patients with pathogenic variants in TRC pathway. The first patient carrying dramatically low level of CAML presented with severe neurological disorder with muscular involvement [19]. Similar clinical symptoms were reported in an individual with heterozygous missense mutations in TRC35 that led to significant reductions in three TRC proteins, including TRC35, BAG6, and UBL4A [7]. The findings of these two patients demonstrate a critical role of the tail-anchored membrane protein insertion in neurological function and diseases. Interestingly, biallelic variants in *ASNA1* gene with a premature stop codon leading to reduced expression on one allele and a mutation resulting in protein misfolding on the other allele had been identified in two siblings who died of a very severe cardiomyopathy in their infancy [23]. Heart muscle disorder was specifically described in this case. However, due to their early death, no neurological symptoms were reported.

It is perhaps not surprising that the critical role for CAML is in neurons, as we previously showed that its highest expression was indeed in the nervous system [24]. CAML deficiency is length dependent, with the largest impact observed in the longest of neurons which innervate the lower legs. Certainly, many cellular processes are stretched to their limit by the extreme distance over which communication and transport must occur, from the cell body to the ends of axons and back. Several human diseases, including ALS and hereditary spastic paraplegia (HSP) primarily impact the longest neurons [25,26]. Importantly, deletion of *Caml* genes from embryonic cortical neurons in vitro did not result in defects in proliferation, survival, or neurite outgrowth. We suspect that culture conditions did not provide the long-distance stresses inherent in motor neurons *in vivo*, thus explaining these differences. Further study using our various mouse models is needed to address this.

Yamamoto et al discovered that a highly conserved stretch of positively charged residues in the N-terminus of CAML form the acceptor site that receives client proteins from Asna1 [27]. The N-terminus is therefore essential for TA insertion, a finding we replicated [12]. Our prior studies also revealed an alternative function of CAML that did not require the N-terminus, even in the absence of TA insertion, in the myc-dependent lymphoblastic lymphoma cells [12]. These cells proliferated at an enormous pace, doubling every 12 hours. Survival was not restored by re-expression of a Caml-deletion mutant lacking the N-terminus, which is required for TA protein insertion, whereas small protein fragment consisting of the C-terminal WRB-binding domain rescued growth and survival of *Caml*-deleted lymphoma cells [12]. We therefore suspect that this aspect of CAML biology depended more on its role in chromosome segregation during mitosis. In the case of motor neurons, our evidence points to the importance of TA protein insertion as the key requirement in the nervous system. We generated transgenic mice that express a mutant form of CAML consisting of the C-terminal 111 amino acid residues without the TA-insertion domain. We crossed this transgene into *ERT2-Cre+;Caml*^*fl/fl*^ mice. Treatment with tamoxifen induced hindlimb weakness identical to that seen in mice lacking this C-terminal Caml mutant, suggesting that N-terminal deletion mutant of CAML was not able to sustain hind limb strength upon loss of the full-length endogenous genes. Together with the finding that deletion of the *Asna1* gene gave a very similar phenotype of hindlimb paralysis in the context of the SLICK-H-Cre neuron specific strain, we believe that TA protein insertion is a key requirement in the nervous system.

TA insertion is unusual in that a large number of C-terminal membrane proteins are able to use it for post-translational delivery into membranes, but only a very limited number of those appear to be completely dependent on the TRC pathway. A broad screen of predicted TA proteins in yeast determined that only two out of 46 proteins (Stx5 and Lam5) were severely affected in mutants lacking components of the GET system [18, 28]. In our study, we noted that TA proteins reacted to CAML deficiency differently. In the primary neuron cultures with roughly 50% CAML reduction, the levels of Emerin and VAPB were significantly decreased. However, in the MEFs with almost full knockout of CAML, Stx5 was dramatically reduced. These results support the previous finding that Stx5 is the most sensitive TA protein and is extremely dependent on TRC pathway [28]. Emerin and VAPB appeared to be less associated with CAML level and are likely to be regulated by a coalition of TRC and other pathways. The cotranslational pathway, which is the main mechanism of the membrane protein insertion mediated by the cytosolic signal recognition particle (SRP) [29] or the unconventional SRP independent pathway[30] could be involved in their regulation. In the specialized cells, like neurons, the membrane insertion of TA proteins may require finer and more intricate systems to sustain their proper functions. Regardless of the differences between MEFs and neurons, it is challenging to know for certain which of the client proteins for ASNA1 and CAML are responsible for maintaining motor neuron survival, and it may in fact depend on a multitude of TA proteins.

Nonetheless, we favor the hypothesis that defects in cellular trafficking are fundamental for this mechanism. TA client protein Stx5, which functions in vesicular trafficking and was shown by Amessou et al to be essential for retrograde trafficking [31], was found to be mislocalized and reduced in overall cellular levels in our analyses of CAML-knockout MEFs. Mislocalization of Stx5 was also reported in patient fibroblasts with the homozygous splice variant c.633 + 4A>G in CAMLG [19]. Though the phenotypes in cultured cortical neurons were attenuated, significantly lower cathepsin D level when *Caml* was deleted (Fig 6C) implicates that retrograde transport is indeed targeted by insufficient CAML. Further, the importance of the TRC pathway in intracellular transport is underscored by the findings of Morgens et al [32], who reported that the retrograde transport small molecule inhibitor retro-2 works by directly inhibiting ASNA1.

In addition to improper trafficking, glycosylation disorders have been closely associated with dysfunction of the TRC pathway [7,19]. Consistently, we observed a largely elevated level of glycosylation in *Caml* null MEFs (Fig 5D). Since glycosylation plays a pivotal role in maintaining proper function in the central and/or peripheral nervous systems, the majority of CDGs, including CAMLG-CDG and TRC35-CDG show neurological symptoms [7,19]. Whether the abnormality in glycosylation is a downstream effect of impaired retrograde trafficking or not, they could directly contribute to neuromuscular phenotype in CAML-deficient mice.

Lysosomes are organelles that contain many hydrolytic enzymes. Therefore, they play critical roles in the degradation and recycling of proteins and carbohydrates. Dysfunctions in lysosomes have been implicated in many neurodegenerative diseases, including FTD and ALS [33]. Abnormalities in the endosomal-autophagic-lysosomal system has been reported to promote the pathogenesis of Alzheimer’s disease [34]. In our study, we reported that CAML loss induces dysfunctional lysosomes. Significantly stronger staining on galectin-3 puncta and LAMP1 in *Caml* null cells highlighted the potential role of CAML in maintaining proper lysosomal function. Interestingly, the enlargements of LAMP1-positive structures had been reported in hereditary spastic paraplegia and other neurodegeneration diseases [20]. It will be important to explore whether the hindlimb movement disorder induced by CAML deficiency shares any common mechanism(s) with those diseases presenting enlarged lysosomes. We must address whether the impairment of the lysosomes is a primary dysfunction or a consequence of the alteration of retrograde transport in the context of insufficient CAML.

Using our unique mouse models, we had revealed that CAML deficiency significantly impaired retrograde transport, glycosylation, and lysosomal function. Although the precise pathomechanism needs further elucidation, the combination of these impairments on ER and Golgi could tremendously jeopardize the neuronal survival, causing severe neuromuscular features on the mice.

Whether these observations on the importance of TA protein insertion for intracellular trafficking, motor neuron survival, and muscular innervation will extend from mice to human diseases remains to be seen in future studies. Our work here clearly indicated that drugging the TRC pathway may lead to severe neuro-muscular dysfunction. It is interesting to note, however, that the most common gene alteration in ALS is also thought to involve vesicle trafficking [35]. The TRC pathway components are highly conserved from yeast to mammals. It is tempting to speculate that partial loss-of-function mutations or haplo-insufficiency in one or more of the human TRC pathway components (WRB, CAML, TRC40/ASNA1, TRC35, Ubl4A, Bag-6/Bat-3/Scythe) may in the future be found to be causative in inherited cases of ALS or other motor neuron disease. Importantly, our work provides a new mouse model for motor neuron loss that may be helpful for investigators who study neuronal survival and those who are investigating new approaches to spinal cord regenerative medicine.

## Materials and methods

### Ethics statement

All mice husbandry and experiments were performed following protocols approved by the Institutional Animal Care and Use Committee (IACUC) of Mayo Clinic (Protocol IACUC A00001777-16-R22 titled as “Unique allele breeding” and Protocol IACUC A00003947-19-R22 titles as “CAML in neuromuscular disease”).

### Animal studies

All animal trials were approved by the Mayo Clinic Institutional Animal Care and Use Committee.

All mice were housed under pathogen-free conditions at 23°C to 25°C with a 12-hour light/ dark cycle and were fed with standard laboratory chow (PicoLab^R^ Rodent Diet 20, Cat#: 0007688, LabDiet, St. Louis, MO, USA) and had free access to water. The number of the mice per cage was ≤5. In-cage shelter was provided for single-housed mice. *Caml*^*fl/fl*^ mice, bearing loxP sites flanking exon 2 of *Caml*, which were generated as previously described[13], were crossed to *ERT2-cre+* transgenic mice and bred on a C57BL/6 genetic background. *rtTA+;Caml-TG+* mice and *Caml* hypomorphic mice were generated by Mayo Transgenic & Knockout Mouse Core. SLICK-H-Cre [STOCK Tg(Thy1-cre/ERT2,-EYFP)HGfng/PyngJ, Strain #:012708] transgenic mice were acquired from The Jackson Laboratory (Bar Harbor, ME, USA). Synapsin-Cre transgenic mice were acquired from Dr. Charles L. Howe at Mayo Clinic. *Asna1*^*fl/fl*^ mice were generated as previously described [17].

Tamoxifen (Sigma-Aldrich, CAS # 10540-29-1) was dissolved in corn oil at a concentration of 18.5 mg/ml. The injection dose was determined by mice body weight. The administration was via intraperitoneal injection.

Doxycycline (Sigma-Aldrich, CAS # 24390-14-5) was dissolved in water containing 1% sucrose at a concentration of 0.2 μg/mL. *rtTA+;Caml-TG+;ERT2-cre+;Caml*^*fl/fl*^ and the control mice were fed with low concentrations of doxycycline in the drinking water one week prior to tamoxifen treatment. They were continuously administered until the experiments ended.

We adopted a scoring system for evaluating mouse models of cerebellar ataxia [36]. Measures include hindlimb clasping (marker of neurodegeneration progression), ledge test (direct measure of coordination), and gait (test of both coordination and muscle function). Each measure is recorded on a scale of 0–3, with 0 representing an absence of the relevant phenotype and 3 representing the most severe manifestation.

### Generation of MEFs and embryonic cortical neuron cells

*Caml*^*fl/fl*^ MEFs were generated from 13.5-day embryos as previously described [11]. Primary cells at passage 1 (p1) were divided into two populations and treated with either vehicle or 2 ng/mL of 4HT for 48 hours and released into normal growth media. In most experiments, unless noted otherwise, cells at passage 3, 4, and 5 (p3, p4 and p5) were used for experiments.

Primary embryonic cortical neurons carrying *ERT2-cre+;Caml*^*fl/fl*^ or *ERT2-cre-;Caml*^*fl/fl*^ were generated from the brains dissected from18-day embryos as previously described [37]. On the next day, 4HT (1 ng/mL) to delete *caml* gene and cytosine arabinofuranoside (2 μM) to prevent glial proliferation were added into cells. Cells were incubated with 4HT for 24 hours and released into normal growth media. The impact of acute deletion of CAML in cortical neurons was assessed on day 10 post-tamoxifen exposure.

### IncuCyte NeuroTrack analysis

Primary mouse cortical neuron cultures were infected with AAV1-synapsin-GFP (Addgene, #50465-AAV1) and seeded on poly-ornithine-coated 96-well TPP (Techno Plastic Products AG, Switzerland) plates in the presence or absence of tamoxifen. The IncuCyte ZOOM live-cell imaging system S3 (Essen BioScience, UK) was used to analyze neurite dynamics starting on day 3. NeuroTrack software was used to quantify living cells’ neurite dynamics. Different parameters were set as detailed previously [38]. Plates were scanned every 4 hours over a 250-hour period using a 20x objective. Two images per well were captured and images were analyzed for neurite length. Each image included 30 ~ 60 neuron cells in average.

### Western blot analyses

Western blot analyses were carried out as previously described [39]. Equal loading was determined using tubulin or actin. Rabbit polyclonal antibodies to human CAML (1–189) have been described previously [12]. Antibodies for VAPB was purchased from Proteintech (14477-1-AP). Antibodies for Emerin was purchased from Proteintech (10351-1-AP). Antibodies for Syntexin 8 (FNab08456) was purchased from www.fn-test.com. Antibodies for Syntexin 5 (ABIN1809664) was purchased from www.antibodies.com. Antibodies for Cathepsin D (ab75852) was purchased from Abcam. Procathepsin D shown in Fig 4A was prepared from the media growing *ERT2-cre-;Caml*^*fl/fl*^ MEFs in the presence or absence of 4HT. Cells were serum starved for overnight before harvesting the media. The dead cells were removed from the growing media prior to concentrating via 10k Da MWCO Vivaspin6 (GE healthcare 28-9322-96). Antibodies for Tubulin (T6793) was purchased from Sigma. Antibodies for Actin (ab149647) was purchased from Abcam. The Precision Plus Protein Dual Color Standards purchased from Bio-Rad (Cat# 610374) was loaded with all targeted proteins on polyacrylamide gel. Membranes were visualized by developing films on Kodak X-OMAT 2000A. All expression data were scanned and measured by Image J 1.53k software (https://imagej.nih.gov/ij/), and then normalized to actin or tubulin.

### Immunofluorescence

Cells were fixed with 4% paraformaldehyde and permeabilized by 0.1% Triton. Cells were blocked with 5% goat serum, 1% glycerol, and 0,1% BSA in PBS (blocking buffer) for 1 hour and incubated with primary antibodies diluted in blocking buffer at 4°C overnight. Antibodies for Syntexin 5 (B-8, sc365124) was purchased from Santa Cruz Technology. Antibodies for CD222 (ab2733) was purchased from Abcam. Antibodies for Galectin 3 (ab2785) was purchased from Abcam. Antibodies for choline acetyltransferase (ab178850) was purchased from Abcam. Antibodies for LAMP1 (ab208943) was purchased from Abcam. Antibodies for GM130 (610822) was purchased from BD Transduction Laboratories. Incubation with Alexa Fluor secondary antibodies (Invitrogen) was performed for 1 hour at room temperature, and the cells were countstained by Hoechst. All samples were mounted with Dako fluorescence mounting medium (S3023). Images were taken using a Leica DIMI microscope or Zeiss confocal laser scanning microscope.

### Histology

4 weeks after 2 doses of 75 mg/kg tamoxifen treatment, control mice (*SLICK-H-Cre*^*-*^*;Caml*^*fl/fl*^ or *SLICK-H-Cre*^*+*^*;Cam*^*+/+*^) or *SLICK-H-Cre*^*+*^*;Caml*^*fl/fl*^ mice were humanely euthanized and immediately perfused with phosphate buffered saline (PBS) to decrease blood contamination. Mice were then perfused with 4% paraformaldehyde. The spinal column from the base of the skull to just caudal to the femurs were dissected out and fixed in 4% paraformaldehyde for overnight at 4°C. The first cut was made between T11 vertebral body and rostral T12 body that corresponds to L2 on the spinal cord. The second cut was made between caudal T13 body and rostral L1 body that corresponds to L5 on the spinal cord. The lumbar region of spinal cord (Segment L2, L3, L4, L5) was isolated from the column segment under the microscope. The tissues were then processed in Leica ASP300S. After processing, the tissues were cut into 2 equal segments. Using forceps, the spinal cord was oriented vertically with the cut end down and embedded in paraffin using Leica EG1150C. We saved the 1^st^ 5-μm section onto slides. Each slide contained 2 consecutive sections from L3-L4 region of the spinal cord was stained with hematoxylin and eosin following standard procedures.

### Lectin staining

Primary MEFs (80–90%confluence) were cultured on 10-well slides in DMEM supplemented with 10%FBS, under 5% CO2, 3% O2 atmosphere. For lectin staining, cells were washed 3 times with HBSS (14025–092, Gibco) Peanut Agglutinin (PNA), Fluorescein (FL-1071) (Vector laboratories, California, USA) were diluted at 5*μ*g/ml in HBSS, applied on MEFs and incubated for 10 minutes at room temperature protected from light. Cells were then washed twice with HBSS and fixed with 4% paraformaldehyde in PBS for 10 minutes at room temperature. Nuclei were stained with Hoechst (in PBS) and cells were mounted with antifade mounting medium (Vectashield H-1000) on glass slides.

### Statistical analysis

All statistics were performed using GraphPad Prism 10 software (GraphPad Software, Inc., La Jolla, CA, USA). Data are expressed as mean ± SEM. *p<0.*05* was considered statistically significant.

## Supporting information

S1 Video Movement of a control mouse and a *rtTA+;Caml-TG+;ERT2-Cre+;Caml*^*fl/fl*^ mouse at 2 weeks post-tamoxifen treatment. (MPG)

S2 Video Movement of two control mice and a *SLICK-H-Cre+;Caml*^*fl/fl*^ mouse at 2 weeks post-tamoxifen treatment. (MOV)

S3 Video Movement of a control mouse and a *SLICK-H-Cre+;Asna1*^*fl/fl*^ mouse at 2 weeks post-tamoxifen treatment. (MOV)

S1 Data Summary statistics for Fig 1D. (PZFX)

S2 Data Summary statistics for Fig 1E. (PZFX)

S3 Data Summary statistics for Fig 1F. (PZFX)

S4 Data Summary statistics for Fig 1G. (PZFX)

S5 Data Summary statistics for Fig 2A. (PZFX)

S6 Data Summary statistics for Fig 2B. (PZFX)

S7 Data Summary statistics for Fig 2C. (PZFX)

S8 Data Summary statistics for Fig 3A. (PZFX)

S9 Data Summary statistics for Fig 3B. (PZFX)

S10 Data Summary statistics for Fig 4A. (PZFX)

S11 Data Summary statistics for Fig 4B. (PZFX)

S12 Data Summary statistics for Fig 4C. (PZFX)

S13 Data Summary statistics for Fig 4D. (PZFX)

S14 Data Summary statistics for Fig 5A. (PZFX)

S15 Data Summary statistics for Fig 5B. (PZFX)

S16 Data Summary statistics for Fig 5C. (PZFX)

S17 Data Summary statistics for Fig 5D. (PZFX)

S18 Data Summary statistics for Fig 6A. (PZFX)

S19 Data Summary statistics for Fig 6B. (PZFX)

S20 Data Summary statistics for Fig 6C. (PZFX)

S21 Data Summary statistics for Fig 6E. (PZFX)
